# Adenosine Prevents TNFα-Induced Decrease in Endothelial Mitochondrial Mass via Activation of eNOS-PGC-1α Regulatory Axis

**DOI:** 10.1371/journal.pone.0098459

**Published:** 2014-06-10

**Authors:** Theodore J. Kalogeris, Christopher Baines, Ronald J. Korthuis

**Affiliations:** 1 Department of Medical Pharmacology and Physiology, University of Missouri, Columbia, Missouri, United States of America; 2 Department of Biomedical Sciences, University of Missouri, Columbia, Missouri, United States of America; 3 Dalton Cardiovascular Research Center, University of Missouri, Columbia, Missouri, United States of America; Albany Medical College, United States of America

## Abstract

We tested whether adenosine, a cytoprotective mediator and trigger of preconditioning, could protect endothelial cells from inflammation-induced deficits in mitochondrial biogenesis and function. We examined this question using human microvascular endothelial cells exposed to TNFα. TNFα produced time and dose-dependent decreases in mitochondrial membrane potential, cellular ATP levels, and mitochondrial mass, preceding an increase in apoptosis. These effects were prevented by co-incubation with adenosine, a nitric oxide (NO) donor, a guanylate cyclase (GC) activator, or a cell-permeant cyclic GMP (cGMP) analog. The effects of adenosine were blocked by a nitric oxide synthase inhibitor, a soluble guanylate cyclase inhibitor, a morpholino antisense oligonucleotide to endothelial nitric oxide synthase (eNOS), or siRNA knockdown of the transcriptional coactivator, PGC-1α. Incubation with exogenous NO, a GC activator, or a cGMP analog reversed the effect of eNOS knockdown, while the effect of NO was blocked by inhibition of GC. The protective effects of NO and cGMP analog were prevented by siRNA to PGC-1α. TNFα also decreased expression of eNOS, cellular NO levels, and PGC-1α expression, which were reversed by adenosine. Exogenous NO, but not adenosine, rescued expression of PGC-1α in cells in which eNOS expression was knocked down by eNOS antisense treatment. Thus, TNFα elicits decreases in endothelial mitochondrial function and mass, and an increase in apoptosis. These effects were reversed by adenosine, an effect mediated by eNOS-synthesized NO, acting via soluble guanylate cyclase/cGMP to activate a mitochondrial biogenesis regulatory program under the control of PGC-1α. These results support the existence of an adenosine-triggered, mito-and cytoprotective mechanism dependent upon an eNOS-PGC-1α regulatory pathway, which acts to preserve endothelial mitochondrial function and mass during inflammatory challenge.

## Introduction

The process of mitochondrial biogenesis–the coordinated orchestration of nuclear and mitochondrial gene expression, mitochondrial protein import, and structural dynamics, so as to optimize cellular mitochondrial function–has recently been proposed as a potentially useful therapeutic target in the protective effects of ischemic preconditioning (IPC) [1]. However, a direct test of the role of mitochondrial biogenesis in IPC has not yet been reported. Although it is known that IPC upregulates mitochondrial biogenesis, as well as cellular pathways mediating its control [1], it is unclear to what extent biogenesis *per se* may be responsible for IPC-elicited protection. Similar uncertainty exists regarding the precise role of mitochondrial biogenesis in mediating other preconditioning strategies, such as ingestion of low-moderate doses of ethanol [2]–[4], or antecedent treatment with hydrogen sulfide [5]–[7], adenosine [4], [8], [9], carbon monoxide [10], [11], isoflurane [12] or exercise training [13], [14]–even though several of these treatments have indeed been found to influence mitochondrial function and/or mass [5]–[7], [10]–[14]. A complicating issue is that under certain conditions, increased mitochondrial mass may in fact, be deleterious [15],[16].

The role of the vascular endothelium as a target for both the injurious effects of IR, as well as the protective effects of preconditioning is well established. Although it is not known to what extent mitochondrial biogenesis in the endothelium might play in these processes, it is reasonable to propose such a role, by virtue of this organelle’s recognized function as a platform for coordination of redox-dependent cell signaling and cell death [8],[17]–[20]. Of more direct relevance, it has been shown that endothelial cells have a reserve mitochondrial bioenergetic capacity that may play a cytoprotective role in the response to stress [21]. However, results from studies in other cell/tissue types are conflicting. It has been shown in several cell types that improvements in mitochondrial reserve capacity and/or function might be explained by increases in mitochondrial mass [22]–[24]. But other studies in heart and skeletal muscle have reported a dissociation between mitochondrial mass and function [15],[16],[25]. Examination of this issue in endothelial cells has not been reported.

Adenosine is an endogenous mediator whose production and release is triggered by various types of cell stress, and which can modulate tissue damage and repair [26]. It has been shown to play an important, early role in triggering the protective effects of ischemic and several types of pharmacologic preconditioning in experimental models of ischemia/reperfusion (I/R) [4],[9],[27]. Increased levels of tissue adenosine appear to be a particularly critical prerequisite for achieving the delayed preconditioned phenotype [2]–[4]. It has been proposed that adenosine may be an initial triggering element in a signaling cascade that is activated by ischemic preconditioning. Although precise details of this cascade are not yet clearly elucidated, it appears that an immediate downstream mediator of adenosine’s protective effect is eNOS-dependent release of nitric oxide (NO) [4],[28]. Nitric oxide, in turn, has been shown to play a critical role in both mitochondrial function and biogenesis [22],[29]–[31], and is known to modulate expression of PGC-1α [32], a key master regulator of both energy metabolism and mitochondrial biogenesis [33]–[35]. Indeed, it was recently demonstrated that TNFα-elicited downregulation of eNOS expression resulted in decreased mitochondrial content in adipose and muscle that could be reversed by administration of NO donors [31]. Taken together, the aforementioned observations suggest the hypothesis that adenosine’s protective effect might be mediated, at least in part, by NO-dependent defense of mitochondrial mass in endothelial cells. To test this possibility, it would first be important to determine 1) the effect of a model pro-inflammatory stressor on indices of mitochondrial function and mass in endothelial cells, 2) whether any such effect can be modulated by adenosine, and 3) whether adenosine-induced protection might be mediated through a NO-dependent mechanism.

The purpose of this study was to address the aforementioned three aims. We have developed a model to examine markers of mitochondrial mass in human microvascular endothelial cells (HMEC-1) challenged with the proinflammatory cytokine, TNFα. In the present studies, we report modulatory effects of adenosine on TNFα-elicited increases in apoptosis, associated with decreased mitochondrial mass and function, and show for the first time, that these effects of adenosine are mediated by activation of an eNOS-PGC-1α regulatory pathway.

## Materials and Methods

### Cell Culture

Human dermal microvascular endothelial cells (HMEC-1) [36] were obtained from the Centers for Disease Control (Atlanta, GA) and maintained in MCDB-131 Media (Sigma-Aldrich, St. Louis, MO) supplemented with 10% heat-inactivated fetal bovine serum (FBS, Atlanta Biological, Atlanta, GA), mouse epidermal growth factor (10 ng/ml, Becton-Dickenson, Bedford, MA), hydrocortisone (1 µg/ml, Sigma-Aldrich), HEPES (10 mM), and pyruvate (1 mM), an atmosphere of 5% CO_2_ at 37°C. Medium was changed every 3–4 days and cells were passaged once per week. For experiments cells were grown in either 100 mm culture dishes, or gelatin-coated glass coverslips and used for experiments at 2–3 days post-confluence.

### Experimental Treatment Protocols

Initial experiments examining the effects of TNFα on apoptosis and mitochondrial mass studies were aimed at defining the time course of the HMEC-1 response to TNFα. For examination of apoptosis, cells on glass coverslips were treated for 4, 8, 12, 24, 48, or 72 h with either HBSS (control) or 1 or 10 ng/ml TNFα dissolved in HBSS. For the 48 and 72 h timepoints, fresh TNFα was added to the cells every 24 h. For measurement of mitochondrial mass, cells in 100 mm dishes were similarly treated. At the indicated time points, cells were harvested for analysis. Based on results of these initial studies, all subsequent experiments were carried out in cells incubated with TNFα, in the presence or absence of other factors, for 48 h. In experiments testing the effects of adenosine (10 µM), (Z)-1-[2-(2-aminoethyl)-N-(2-ammonioethyl) amino] diazen-1-ium-1, 2-diolate (detaNO, 10–1000 µM), N5-(1-iminoethyl)-L-ornithine, dihydrochloride (L-NIO,100 µM), 1H-(1,2,4)-oxadiazolo-[4,3-alpha]-quinozalin-1-one (ODQ, 30 µM), 3-(5′-hydroxymethyl-2′-furyl)-1-benzyl indazole (YC-1, 100 µM), and 8-bromo-cyclic GMP (8-Br-cGMP, 500 µM), each was added to cells immediately prior to TNFα both at the start of the experiment, and again at 24 h. *eNOS antisense and PGC-1α siRNA experiments:* The role of endogenous eNOS in mediating the effect of adenosine on TNFα-induced decrease in mitochondrial mass was tested using cells that had been transfected with a morpholino antisense oligomer construct (Gene Tools, Philomath, OR) to eNOS. Sequence of the antisense construct (NOS3) was AAGATAGTGGACGAGGCTTGACTCA; this was tested against both invert (3SON: ACTCAGTTCGGAGCAGGTGATAGAA) and mis-paired (NOS3 mis: AAGTTACTGGACCAGGCTAGAGTCA) antisense negative control oligos. Cells were transfected at 80% confluence using Endo-Porter reagent (Gene Tools), beginning 48 h prior to experiments. At 24 h, Endo-Porter-containing media was gently washed out and replaced with fresh media. Experimental treatments were begun at 48 h post-transfection.

We used an siRNA to PGC-1α (sc-38884, Santa Cruz Biotechnologies, SantaCruz, CA) to examine the role of this regulatory factor in mediating both adenosine- and NO-induced preservation of mitochondrial mass during exposure to TNFα. Similar to the eNOS antisense studies, cells were transfected with siRNA or control constructs at about 80% confluency, 48 h prior to initiation of experiments, according to the manufacturer’s instructions. Knockdown efficacies for both eNOS and PGC-1α were determined by immunoblotting at 48 h after transfection.

### Endothelial Apoptosis

We evaluated the time-dependent effect of TNFα dose on apoptosis in HMEC-1 cells as previously described [37]. Twenty-four hours prior to experiment, cells were seeded at a density of 10^5^ cells/ml on gelatin-coated, 12 mm circular glass cover slips. Cells were incubated with or without TNFα (1 or 10 ng/ml) for 4–72 h. They were then washed with PBS and fixed for 15 min in ice-cold 4% paraformaldehyde, washed again with PBS and fixed for 1 h at −20°C with ice-cold 70% ethanol. Coverslips were mounted on glass slides using Vectashield mounting medium containing 4-6-diamidino-2-phenylindole (DAPI, Vector Laboratories, Burlingame, CA). Cells were viewed and counted at 40X magnification using a Nikon Eclipse E600 fluorescence microscope. On each slide, at least 200 apoptotic and total cells were counted in six random fields of view. Cells were judged to be apoptotic on the basis of clearly observed chromatin condensation, nuclear fragmentation, and apoptotic bodies [38]. In separate studies, we also examined a second indicator of apoptosis, i.e. activation via proteolytic cleavage of the effector caspase, caspase-3, by western blot, using antibody directed against human caspase-3 (Cell Signaling Technology, Danvers, MA).

### Mitochondrial Membrane Potential

Mitochondria membrane potential was determined using the cell permeant, cationic fluorescent dye, tetramethyl rhodamine, methyl ester (TMRM) (Invitrogen, Grand Island, NY), fluorescence of which is dependent on mitochondrial polarization. Cells were washed with HBSS, then divided into four equal aliquots; one aliquot was resuspended in serum-free media containing TMRM (150 nM), and the second in media containing a similar volume of DMSO (TMRM diluent), the latter was used to correct values obtained from the dye-loaded cells for any possible autofluorescence. Cells were incubated for 20 min in the dark to facilitate loading of the fluorophore. Dye- or diluent-loaded cells were centrifuged at 500 g for 5 min, then resuspended in EIB+150 nM TMRM to maintain the equilibrium distribution of the dye. Aliquots of cell suspension were then transferred to a black 96-well plate and TMRM fluorescence was measured at 548 nm (excitation) and 573 nm (emission) in a plate reader. The other two aliquots were used to obtain a value for total mitochondrial mass, using the cell-permeant, mitochondrial-selective fluorescent dye, Mitotracker Green (MTG, Invitrogen), whose uptake and retention is independent of the state of mitochondrial polarization. Cells were loaded with MTG (150 nM) or DMSO for 15 min in the dark, then fluorescence at 485 nm (excitation) and 528 nm (emission) was measured. Results were expressed as the ratio of fluorescence signal from TMRM to MTG, each corrected for the respective values for DMSO.

### Measurement of Cell Nitric Oxide

At the conclusion of experimental treatment, cells in 6-well plates were washed free of media with HBSS, then loaded with the fluorescent dye, 4-amino-5-methylamino-2′, 7′-difluorescein (DAF-FM, 5 µM, diacetate, Molecular Probes, Invitrogen) in HBSS+10 mM HEPES for 30 min. Cells were washed free of unincorporated dye, incubated a further 15 min in fresh buffer, then washed and harvested in PBS by gentle scraping. For each sample, separate aliquots were then either lysed and assayed for protein content or subjected to fluorescence measurement (485 nm exc., 528 nm emm.) Fluorescence measurements were normalized to protein content, and cell NO in response to a given treatment was expressed as the percent of control values. In addition, for each treatment, separate wells were also treated with the specific NO scavenger, 2-Phenyl-4,4,5,5-tetramethylimidazoline-1-oxyl 3-oxide (PTIO, 1 mM, Sigma-Aldrich, St. Louis, MO) in order to further correct fluorescence values for non-NO-specific fluorescence [39]. The concentration of PTIO used (1 mM) was determined from preliminary experiments conducted in both cell-free, detaNO (100 µM)-containing buffer (HBSS+HEPES, pH 7.4), and in cells incubated with exogenous detaNO in which the level of PTIO was titrated until fluorescence values were rendered undetectable.

### Measurement of Cellular ATP

Cells in 6-well plates were washed with, then scraped and suspended in 1 ml ice-cold PBS. An equal volume (1 ml) of 10% (w/v) trichloroacetic acid (TCA) containing 4 mM EDTA was added, and the cells were lysed by sonication. The lysate was split into two equal aliquots. To one of these aliquots was added a known amount of ATP standard, to the other an equivalent volume of PBS. To remove the TCA, lysates were transferred to stoppered, 15 ml Corex extraction tubes and subjected to three rounds of extraction using water-saturated diethyl ether. Phase separation was obtained via centrifugation at 3000×g for 10 min, and after the final extraction and removal of the organic phase, residual ether was removed by gentle bubbling of N_2_ through the aqueous phase for 10–15 min. A 10 µl aliquot of extract sample (diluted if necessary) was mixed with luciferase reaction buffer, pH 8.0 (Invitrogen/Molecular Probes #A22066) and light emission at 560 nm was measured on a luminescent plate reader. Values were corrected for background fluorescence and recovery of ATP through the extraction procedure (based on the value obtained from the lysate aliquot with added ATP standard; recoveries ranged from 95–98%), and ATP was quantified using a standard curve.

### Measurement of Mitochondrial Mass

We used several methods to determine mitochondrial mass: uptake of mitotracker green (MTG), quantitation of mitochondrial and nuclear DNA using a real-time PCR assay, measurements of citrate synthase activity, and western blot analysis of several key mitochondrial proteins.

#### Mitotracker green assay

We developed a plate assay using MTG. Cells plated in 100 mm dishes were treated as described below. They were gently washed, twice with HBSS, then incubated for 30 min at 37°C with prewarmed, serum-free medium containing 150 nM mitotracker green (MTG, Invitrogen, Grand Island, NY). At the end of the incubation period, cells were washed with PBS, then gently scraped from the plate in 0.3 ml PBS. The cell suspension was gently mixed, then divided into two aliquots: 0.2 ml was transferred to a black assay plate for direct measurement of MTG fluorescence, and the remaining 0.1 ml was used for assay of total protein. Fluorescence was measured using a fluorescence plate reader at 490 nm (Exc.) and 516 nm (Em.). Protein was measured using the D_c_ assay (Biorad, Hercules, CA). Mitochondrial mass was expressed as the ratio of MTG fluorescence to total protein.

#### Isolation of total cellular DNA and quantitation of mitochondrial and nuclear DNA

After treatments, cells were washed with ice-cold PBS, then harvested by scraping into 1 ml PBS. They were centrifuged at 500 g for 10 min at 4°C, the supernatant discarded, and the cell pellet was resuspended in 400 µl lysis buffer (10 mM Tris, pH 8.0, 25 mM EDTA, 100 mM NaCl, 1% SDS, and 3 U/ml proteinase K (Thermo Fisher Scientific, Waltham, MA). Samples were incubated with gentle agitation for 5 h at 55°C. When digestion was complete, samples were incubated with DNase-free RNase (0.8 µg/ml, Roche, Diagnostics, Indianapolis, IN) for 30–45 min. They were then subjected to extraction using phenol/chloroform/isoamyl alcohol pH 8.0. Phase separation was obtained using phase-lock gel tubes (5-Prime, Inc., Gaithersburg, MD), DNA was precipitated using isopropanol, washed with 100% ethanol, then the purified DNA pellet was resuspended in Tris-EDTA (TE) buffer, pH 8.0. DNA concentration was determined after mixing an aliquot of sample with Hoechst 33258 bisbenzamide dye (Sigma-Aldrich) and measuring fluorescence (360 nm Exc., 460 nm Em.) in a fluorescence plate reader, using purified calf thymus DNA as standard.

We performed qPCR using an assay developed using primers (Eurofins MWG Operon, Huntsville, AL) for 12s mitochondrial DNA (mtDNA, fwd: ATTTCGTGCCAGCCACCGCGG; rev: GGCTACACCTTGACCTAACGT) and 18s nuclear DNA (nDNA, fwd: GGAATAATGGAATAGGACCGCG; rev: GGACATCTAAGGGCATCACAG), using SYBR Green detection on an IQ5 real time cycler (Biorad). Amplification efficiencies for both primer sets were determined from calibration curves derived from HMEC-1 total DNA obtained in an identical manner as in these experiments; these were 94.4±4.7% (R^2^ = 0.994, slope = −3.464, y-intercept = 3.048), and 89.7±5.4% (R^2^ = 0.990, slope = −3.595, y-intercept = 9.428) for mitochondrial and nuclear primer sets, respectively. Amount of mtDNA (relative to sham condition) was normalized to reference nDNA using the 2^−ΔΔCt^ (Livak) method [40]; data for this latter value are reported herein as the ratio of mitochondrial to nuclear DNA (mtDNA/nDNA).

### Citrate Synthase Activity

We measured citrate synthase activity in whole cell lysates using a commercially-available, colorimetric assay kit (Sigma #CS0720) according to manufacturer’s recommendations. Supernatants from 20,000×g lysates from all experiments were assayed for protein, then flash-frozen in liquid N_2_, and stored at −80°C for no more than 5 days before performing the assay. Activity was expressed as µmol ml^−1 ^g protein^−1^.

### Extraction of Cellular Proteins and Immunoblotting

Cells were washed in ice-cold PBS, then lysed by sonication in modified RIPA buffer (150 mM NaCl, 50 mM Tris, pH 8.0, 1% Triton X-100, 0.05% SDS, 1 mM PMSF and 10 µl/ml protease inhibitor cocktail (#P8340, Sigma-Aldrich). Lysates were centrifuged at 12,000×g for 10 min, and equal amounts of total protein were subjected to SDS-PAGE followed by western blotting and immuno-detection, using primary antibodies against PGC-1α, Mfn-2, Nrf-2 (Cell Signaling Technology, Danvers, MA), eNOS (Pharmingen/BD Biosciences, San Diego, CA), porin (CalBiochem/EMD Millipore, San Diego, CA), subunit 2 of cytochrome oxidase-IV (cox-IV) (Invitrogen, Grand Island, NY), and GAPDH (Chemicon, Temecula, CA). After incubation with HRP-coupled, secondary antibody (Cell Signaling Technology), blots were developed using chemiluminescent detection (Super West Pico, Pierce/Thermo-Fisher Scientific, Rockford, IL), and semiquantitative densitometric analysis of detected signals was conducted using NIH ImageJ.

### Statistical Analysis

Except where otherwise noted (ATP levels, Tables, 1 & 2), all values herein are reported as mean ± S.E.M.; number of repetitions of each experiment are detailed in the figure legends. Data were analyzed by either one-way or two-way ANOVA with multiple comparisons using a multiple general linear model, or by t-test. Criterion for statistically significant differences was defined as p<0.05.

## Results

### Adenosine Prevents TNFα-induced Mitochondrial Dysfunction and Apoptosis in HMEC-1 Cells

**Figure 1:**
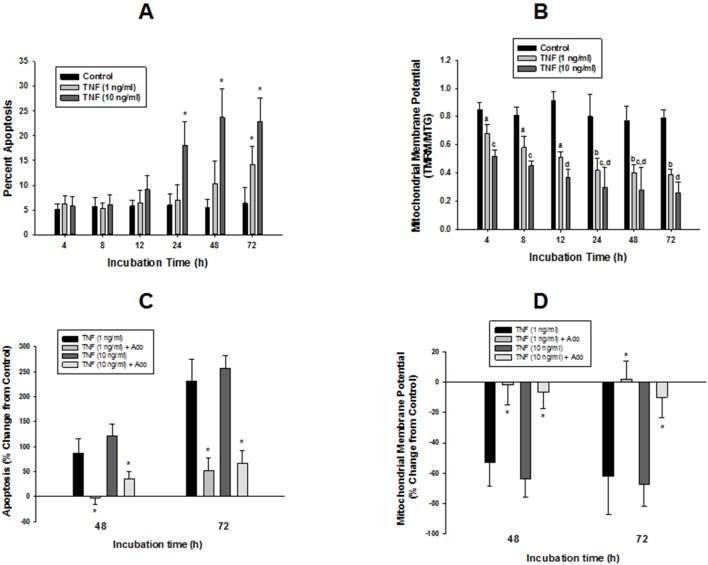
Time and TNFα dose-dependent changes in HMEC-1 apoptosis and mitochondrial membrane potential (Ψ), and modulation of TNFα effects by adenosine. (**A**) HMEC-1 cells on gelatin-coated glass cover slips were incubated with TNFα at 1 or 10 ng/ml for the indicated period. At each time point, apoptotic cells were fixed, mounted, and stained with DAPI, then counted as described in Methods; apoptosis calculated as a percentage of total cells in 6 fields of view, with 200 cells counted per 40X field. Data analyzed by two-way ANOVA with multiple comparisons using a general linear model. Experiments were repeated 3–4 times per treatment group. At a given time of incubation, * indicates significantly different from respective time point control, P<0.01. (**B**) HMEC-1 cells in 24-well plates were incubated with TNFα for the indicated times, loaded with TMRM or MTG dyes, then harvested for measurement of Ψ or total mitochondrial mass, respectively, as described in Methods. Data are means ± SEM for 8 replicates for each treatment/time combination, repeated 4 separate times. Data were analyzed as described for panel (A). All TNFα values were significantly different from their respective controls at each time point, differing letters denote significant TNFα dose effects, P<0.05. (**C, D**) Attenuation of TNFα effects on apoptosis and Ψ, respectively, by adenosine. Cells set up as described for panels (A) & (B) were incubated with or without TNFα (1 or 10 ng/ml), with or without co-incubation with adenosine (Ado, 10 uM) for either 48 or 72 h, then apoptosis or Ψ were measured. Results are expressed as the % change from the respective time point controls. Data are means ± SEM for 4 separate repititions of each experiment. At both time points, all TNFα values were significantly different from respective control values (P<0.001), apoptosis values in response to Ado+TNFα were significantly higher than control at 48 h (TNFα, 10 ng/ml) and at 72 h (both doses of TNFα), p<0.05. * denote significant attenuation of TNFα-induced effect at each time point in response to Ado (P<0.001).

The effect of incubation time and dose of TNFα on endothelial apoptosis are shown in Figure 1A. In all experiments, the proportion of apoptotic cells under control conditions ranged from 4.5 to 6%. TNFα produced a time and dose-dependent increase in endothelial apoptosis. At 1 ng/ml, TNFα’s effect was negligible until 48 h of incubation, and was significantly increased to 14.2±3.7% by 72 h. A ten-fold higher dose of TNFα (10 ng/ml) elicited an earlier apoptotic response: at this higher dose, an trend toward increasing apoptosis was first observed by 12 h, and was significantly elevated to 18.1±4.8% by 24 h, and peaked at 23.7±5.8% by 48 h.

Mitochondrial membrane potential showed a significant decrease that was dependent on TNFα dose, and time-dependent up to 24 h (Figure 1B). Cellular ATP levels showed little response to TNFα from 4–12 h, but showed but significant decreases to between 83–88% of control levels from 24–72 h (Table 1).

**Table 1:** Effect of TNFα dose and time of incubation on cellular ATP levels.

	Incubation Time (h)					
Treatment	4	8	12	24	48	72
**Control**	12.8±1.1	11.2±1.9	11.8±2.0	12.4±1.4	12.4±0.6	11.9±1.1
**TNFα, 1 ng/ml**	11.8±0.5	10.1±1.4	10.4±1.3	10.6±0.4*	10.9±0.2*	9.9±0.6*
**TNFα, 10 ng/ml**	11.5±1.4	10.3±1.8	10.2±2.0	10.7±0.1*	10.3±0.3*	10.0±0.7*

HMEC-1 cells were incubated in the presence or absence of TNFα for the indicated durations, then lysed and total ATP levels (nmol/10^6^ cells) were measured as described in Methods. Values are mean ± S.D. from 4 separate experiments. *denotes significantly different from respective control value.

Co-incubation of cells with adenosine blocked the effects of both low and high doses of TNFα on apoptosis, mitochondrial membrane potential, and cellular ATP levels (Figures 1C, 1D, Table 2). In order to minimize the potential confounding factor of cell death in measurements of MTG uptake, all subsequent studies on mitochondrial mass were conducted using TNFα at 1 ng/ml for 48 h, since this time and dose combination resulted in no significant rise in apoptosis (Figure 1A).

**Table 2:** Cellular ATP levels after incubation with TNFα in the presence or absence of adenosine.

	Incubation Time (h)		
Treatment	24	48	72
Control	11.4±0.9	12.5±0.8	11.6±1.3
TNFα (1 ng/ml)	9.6±0.1*	10.5±0.8*	9.3±0.7*
Ado (10 µM) + TNFα	10.8±0.7	11.7±1.0	12.1±1.8

HMEC-1 cells were incubated in the presence or absence of TNFα or TNFα+Ado (adenosine) for the indicated durations, then lysed and total ATP levels (nmol/10^6^ cells) were measured as described in Methods. Values are means ± S.D. from 3 separate experiments. *denotes significantly different from respective control value.

### TNFα Elicits a Time-dependent Decrease in Mitochondrial Mass

**Figure 2:**
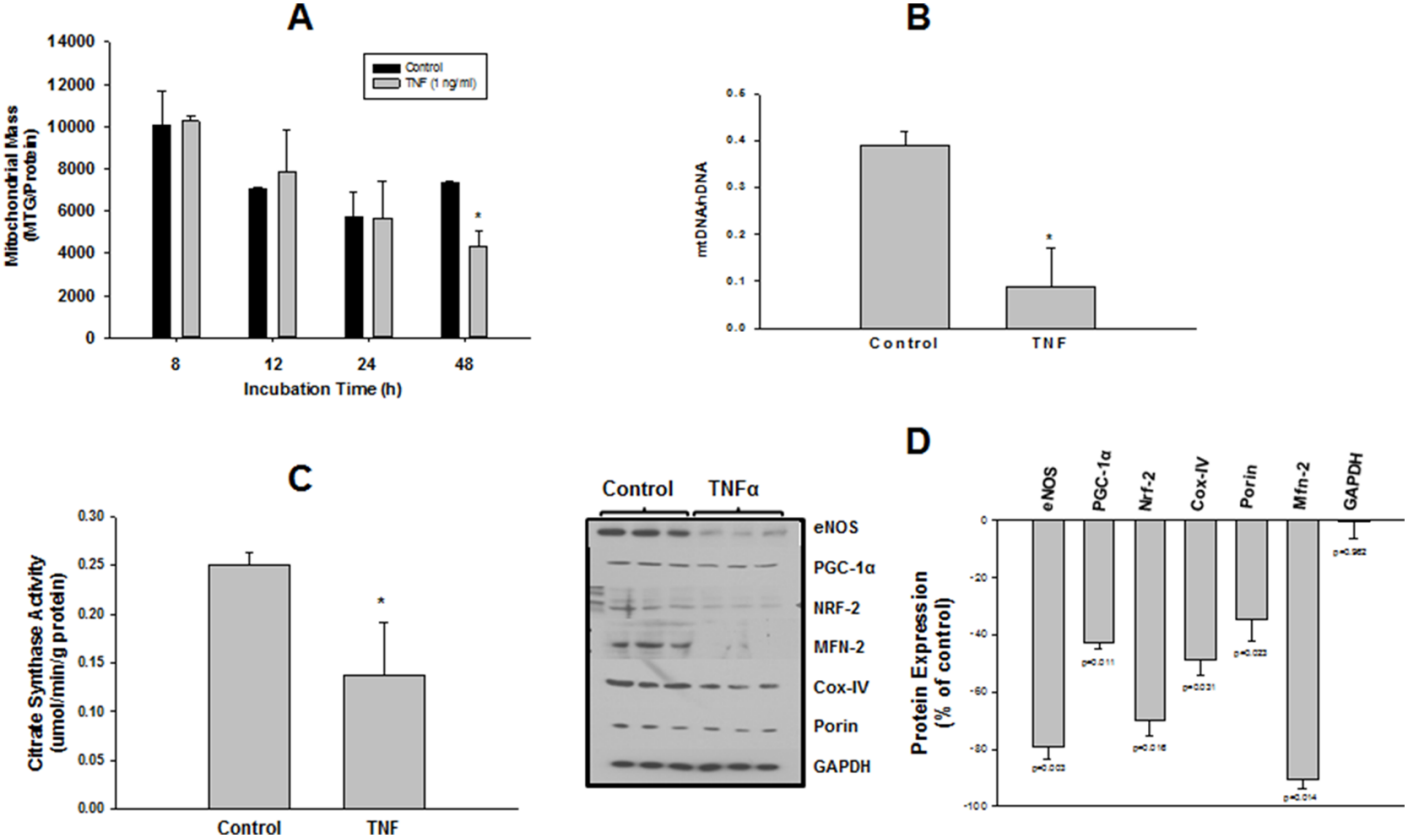
Effects of TNFα on markers of mitochondrial mass and expression of several effector proteins for mitochondrial biogenesis. In separate experiments, HMEC-1 cells in 100 cm plates were incubated with TNFα (1 ng/ml), then 4 separate indices of mitochondrial content were examined. (**A**) Time course of effect of TNFα on mitochondrial mass as measured using a fluorescent plate assay for uptake of MTG dye. At the conclusion of TNFα incubation period, cells were loaded with MTG as described in Methods, then harvested and equal aliquots were separately analyzed for MTG fluorescence and total protein. Mitochondrial mass was expressed as MTG fluorescence, normalized for protein content. Experiments were repeated 3–4 times per time point. * denotes significant decrease in apparent mass compared with respective time point control (P<0.0001). (**B**) Cells prepared as described for panel (A) were incubated in the presence or absence of TNFα (1 ng/ml) for 48 h. Cells were harvested and total DNA was isolated, purified, and subjected to quantitative PCR as described in Methods, using primer sets for both nuclear and mitochondrial DNA (nDNA and mtDNA, respectively). Values are expressed as the mtDNA/nDNA ratio, for 5 separate replications of the experiment. * denotes significant difference from control, p<0.01). (**C**) Cells prepared and treated as described for panel (B) were harvested and lysates were analyzed for citrate synthase activity as described in Methods. Values summarize the results of 4 separate experiments, * denotes significant difference from control, p<0.05. (**D**) Cells prepared and treated as described for panel (B) were harvested and lysates were subjected to SDS-PAGE of equal amounts of lysate protein, followed by western blot anlaysis of the indicated proteins. Left-hand panel shows representative blot from among 3 separate experiments, right-hand panel shows semi-quantitative analysis of band density from the full dataset from all experiments. Except for GAPDH, TNFα elicited significantly decreased expression of all proteins examined, p values for each are shown in the figure.

We observed a time-dependent decrease in MTG fluorescence that was similar in both control and TNFα-treated cells through 24 h of incubation. However, by 48 h, TNFα elicited a 40–45% decrease in fluorescence compared with control which was statistically significant (Figure 2A). This was confirmed by significant, TNFα-induced decreases in mtDNA/nDNA (46%) (Figure 2B) and citrate synthase activity (56%) (Figure 2C). Western blot analysis of several key mitochondrial markers (Mfn-2, porin, and the mitochondrially-encoded subunit 2 of cox-IV) also showed significant decreases in expression in response to 48 h exposure to 1 ng/ml TNFα, with the most striking effect on Mfn-2, whose expression was decreased by over 90% (Figure 2D). TNFα also decreased expression of eNOS, Nrf-2, and PGC-1α (Figure 2D).

### Adenosine Reverses the Effect of TNFα on Mitochondrial Mass and eNOS and PGC-1α Expression

**Figure 3:**
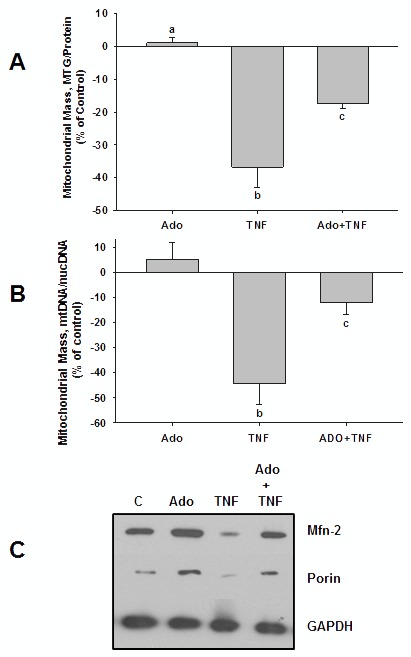
Modulating effect of adenosine (Ado) on TNFα-induced decrease in markers of mitochondrial mass. (**A**) HMEC-1 cells in 100 cm dishes were incubated for 48 h with TNFα (1 ng/ml) in the presence or absence of Ado (10 µM), loaded with MTG, then harvested and MTG fluorescence and protein concentraiton were measured. MTG fluorescence was normalized to protein content; results are expressed as % of control. Experiment was repeated 4 times per group. Both TNFα and Ado+TNFα groups were significantly different from control (p<0.001), differing letters denote significant, between-group differences, p<0.01. (**B**) Total DNA isolated from cells prepared and treated as described for panel (A) was subjected to analysis by qPCR to obtain mtDNA/nDNA ratios. Experiment was repeated 5–6 times per group. Denoting of statistical differences are as described for panel (A). (**C**) Mfn-2, porin, and GAPDH expression in cells prepared and treated as described for panel (A), then lysed and subjected to SDS-PAGE followed by western blot. Figure shows representative blot from 3 separate experiments for each group.

**Figure 4:**
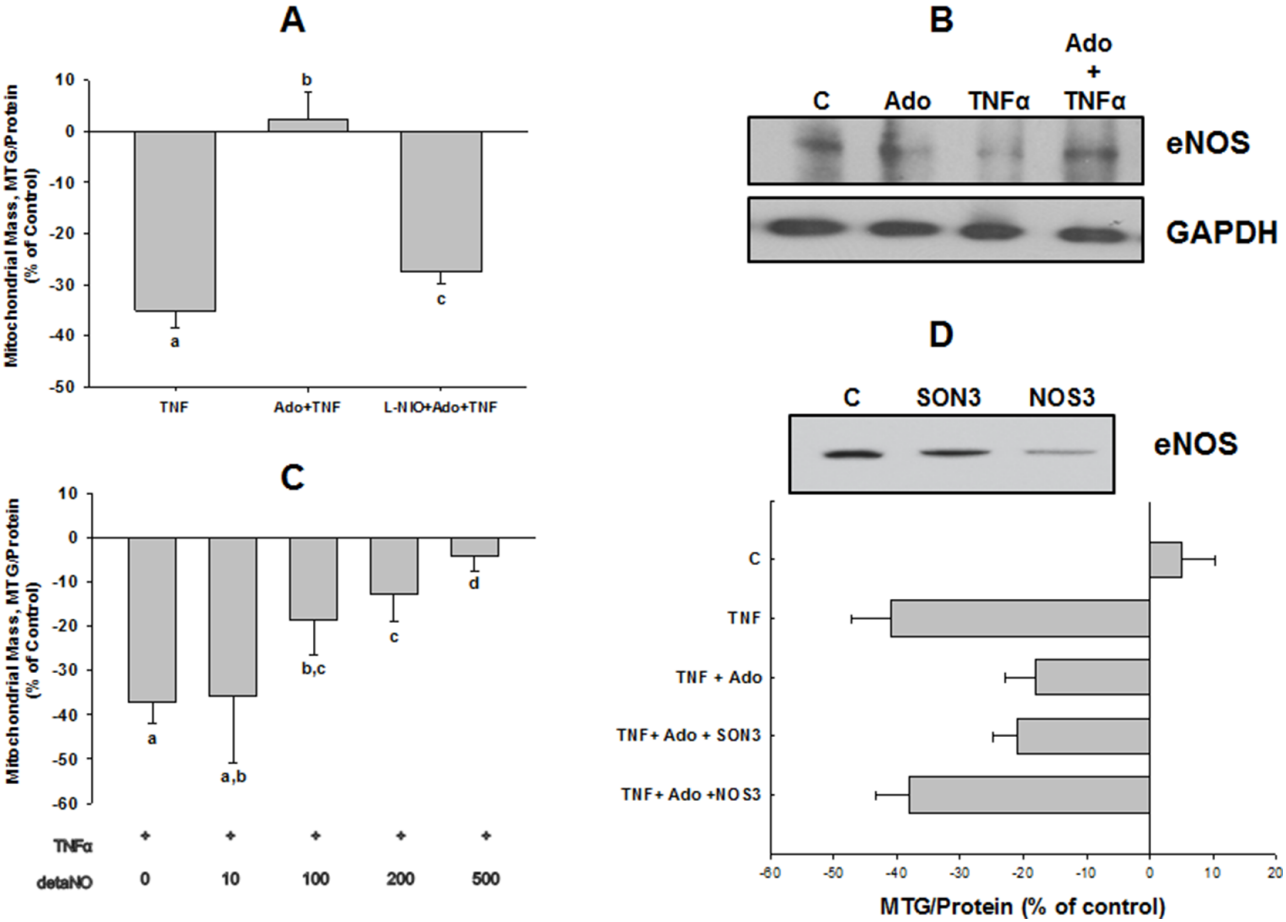
Effect of adenosine (Ado) is mediated by eNOS/NO-dependent mechanism. (**A**) Cells were prepared and treated as described for Figure 3, except that in one group, cells were preincubated for 15 min with L-NIO (100 µM) prior to addition of Ado and TNFα. Mitochondrial mass was assayed using MTG fluorescence as described for previous figures. Values reported are from 3 separate replications of the experiment per group, differing letters denote significant between-group differences, P<0.05. (**B**) Western blot of total eNOS expression in response to TNFα vs. Ado+TNFα; blot shown is from the same experiment shown in Figure 3B. (**C**) Cells were incubated with TNFα in the presence or absence of graded concentrations of the NO donor, detaNO, followed by measurement of MTG fluorescence. Differing letters denote significant dose-dependent differences (p<0.05). Experiment was repeated 4 times. (**D**) Upper panel: western blot of HMEC-1 total eNOS expression, 48 h after transfection with either morpholino eNOS antisense (NOS3) or invert control (SON3) oligonucleotides. Lower panel: MTG fluorescence in cells treated with TNFα±Ado in either non-transfected cells or cells transfected with control or eNOS antisense morpholino oligos. Experiment was repeated 4 times per group, differing letters denote significant between-group differences (p<0.01).

Co-incubation of cells with adenosine (10 µM) attenuated the effect of TNFα on MTG fluorescence (Figure 3A) and on mtDNA/nDNA (Figure 3B) by 53% and 41%, respectively, and completely reversed the effects of TNFα on expression of mitochondrial markers, porin and Mfn-2 (Figure 3C), as well as eNOS (Figure 4B), and PGC-1α (Figure 6B). Although adenosine alone appeared to increase the expression of both porin and Mfn-2 (Figure 3C), we observed no significant effect of adenosine alone on MTG fluorescence (Figures 3 & 8). Adenosine also had no significant effect on mtDNA/nDNA (0.43±0.06, compared with 0.39±0.03 for control, p = 0.08). Collectively, these results suggest that adenosine’s effect may be to prevent TNFα-induced dysfunction in cellular mechanisms controlling mitochondrial function and biogenesis. The dose of adenosine used in these studies (10 µM) is within the range of physiological plasma levels under conditions of stress [41].

**Figure 5:**
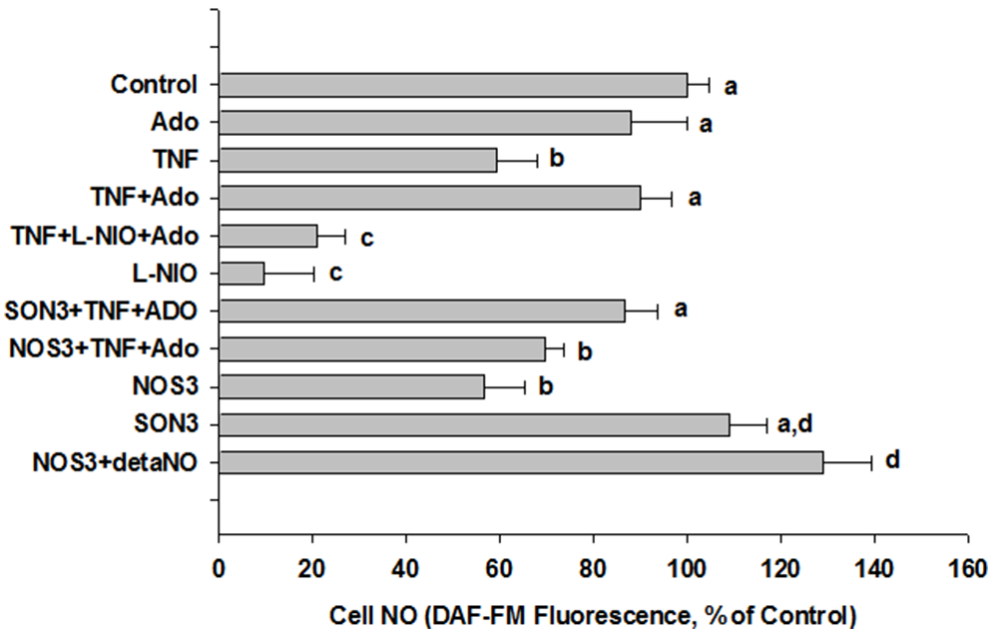
TNFα decreases cell NO, and reversal of this effect by adenosine is eNOS-dependent. HMEC were plated in 6-well plates, then treated for 48 h as described for Fig. 4. Prior to treatment, some cells were transfected with morpholino-antisense or control oligos. Cells were washed free of media then loaded in the dark for 30 min with DAF-FM diacetate (5 µM) in HBSS+10 mM HEPES. Cells were further washed free of DAF-FM-containing buffer, and incubated a further 15 min. They were then gently scraped form the plate and separate aliquots were taken for assay of fluorescence and protein content. All fluorescence measurements were normalized to protein concentration, and the percent change in cellular fluorescence was calculated. Values are means ± S.E.M. for 4 separate experiments of 3 replicates each for each experimental condition except NOS3 (eNOS antisense) + detaNO for which data are for 3 separate experiments. Differing letters denote significant, between-group differences, p<0.05.

**Figure 6:**
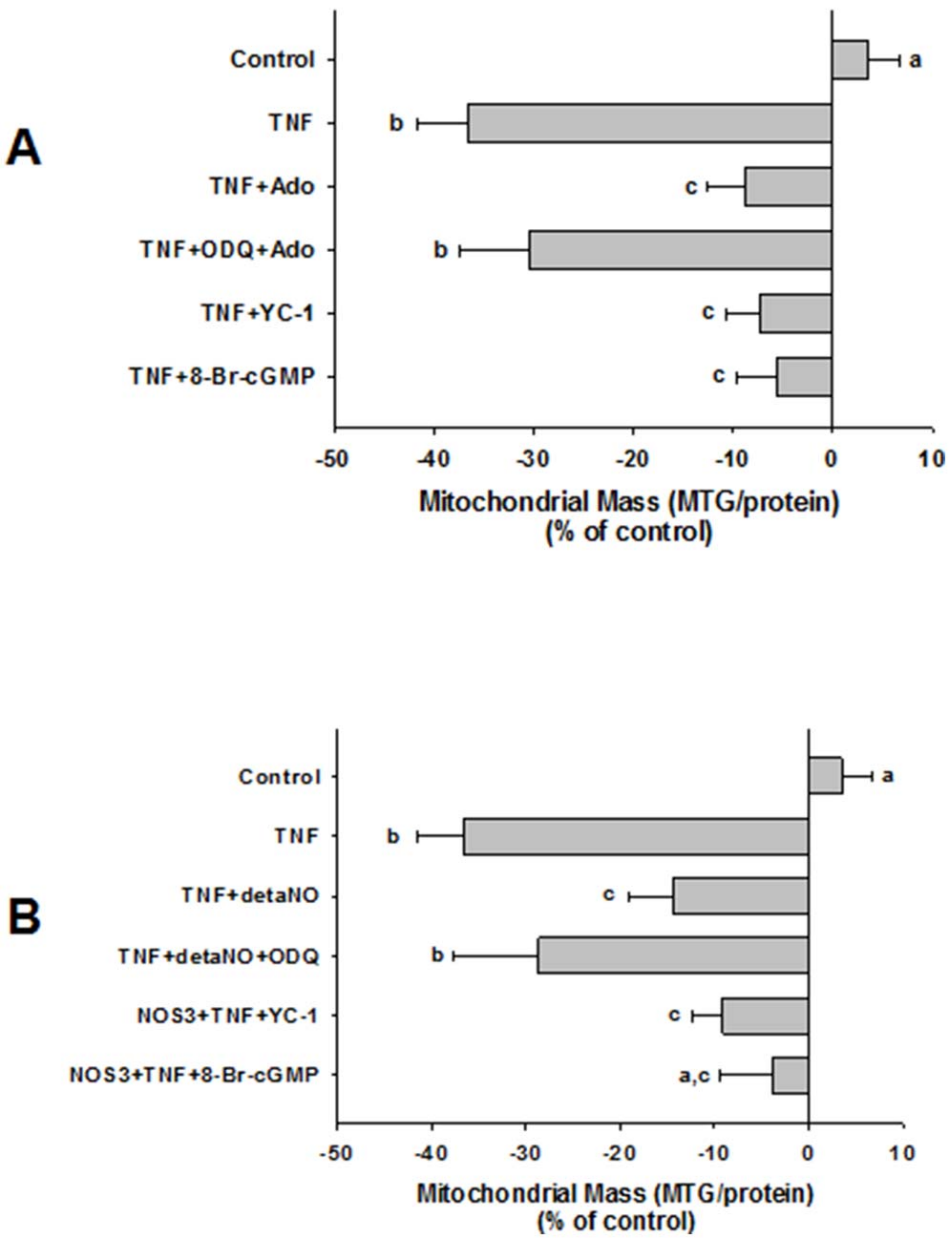
Effect of adenosine/NO is mediated by a sGC/cGMP-dependent mechanism. Both sets of experiments (A, B) were repeated 3 times. (**A**) Cells in 100 cm dishes were incubated for 48 h with TNFα±Ado, in the presence or absence of sGC inhibitor, ODQ (30 µM), sGC agonist, YC-1 (100 µM) or cGMP analog, 8-Br-cGMP (500 µM). Mitochondrial mass measured using MTG fluorescence. Differing letters denote significant between-group differences, p<0.01. (**B**) Cells (non-transfected, or transfected with NOS3 or SON3 morpholino oligos to eNOS) were incubated for 48 h with TNFα±detaNO (100 nM) in the presence or absence of ODQ, YC-1, or 8-Br-cGMP, then MTG fluorescence was measured. Differeing letters denote significant between-group differences, p<0.05.

### eNOS and NO Mediate Adenosine’s Reversal of TNFα-induced Decrease in Mitochondrial Content

Adenosine reversed TNFα-induced decreases in both eNOS expression (Figure 4B) as well as mitochondrial mass. In view of previous studies linking eNOS activity to mitochondrial biogenesis [22],[29]–[31], and our own preliminary results showing adenosine-induced phosphorylation of eNOS at Ser1177, consistent with upregulation of eNOS activity (unpublished observations), we next tested the hypothesis that adenosine’s effect was mediated by eNOS-dependent NO release. First, whereas adenosine completely reversed the effect of TNFα, co-incubation with the NOS inhibitor, N5-(1-iminoethyl)-L-ornithine, dihydrochloride (L-NIO) blocked adenosine’s effect (Figure 4A). Second, TNFα’s effect on MTG fluorescence was reversed in a dose-dependent manner by the NONOate NO-donor compound, detaNO (Figure 4C). The minimum effective dose of detaNO was 100 nM, essentially complete reversal of TNFα’s effect was observed at 500 nM. DetaNO at a concentration of 100 nM releases a maximum of 200 nM NO, a concentration within the physiological range for NO levels in tissue (10–450 nM) [42]. Since the release of 2 moles NO per mole detaNO is only a theoretical maximum [43], it is possible that the effective concentration of NO in our system may have been lower than 200 nM.

These results suggest that adenosine blocks TNFα-induced loss of mitochondrial mass by preventing TNFα-induced decrease in expression of eNOS. However, because L-NIO inhibits all NOS isoforms, we tested the role of endogenous eNOS in cells by knocking down its expression using a specific, morpholino eNOS antisense oligomer. Compared with control cells, the antisense construct (NOS3) reduced eNOS expression by 82–90% whereas the invert (SON3) and mis-paired (NOS3mis, data not shown) controls had no effect (Figure 4D). Using the MTG fluorescence assay for mitochondrial mass, the NOS3 antisense oligo completely blocked the modulating effect of adenosine on TNFα-induced reduction in mitochondrial content (Figure 4D). In control experiments, we found that L-NIO alone produced a decrease in mitochondrial mass that was slightly greater than that produced by eNOS knockdown, but the difference between these two treatments was not statistically significant (Figure 8). These results would suggest that inhibition of eNOS is sufficient to affect mitochondrial mass in HMEC-1 cells.

The above results strongly suggest a critical role for a deficit in eNOS-mediated NO release in the explaining the negative effects of TNFα on mitochondrial mass and function, as well as prevention of this deficit by adenosine. In order to further test this hypothesis, we measured NO levels in response to TNFα, with or without adenosine, L-NIO, or eNOS knockdown, using a fluorescent assay developed using DAF-FM dye (Figure 5). Because use of this dye can be subject to artifactual results due to nonspecific oxidative reactions with non-NO factors [39], values under all conditions were corrected by subtracting out this nonspecific fluorescence using the NO scavenger, PTIO, added to identically treated parallel wells, 10 min prior to a given treatment. For example, non-PTIO-inhibitable (i.e. non-NO-attributable) fluorescence accounted for about 18–25% of raw fluorescence values in control cells. The absolute amount of non-NO-attributable fluorescence was similar across all treatments, but the percentage correction was higher in cells treated with TNFα, L-NIO, or transfected with eNOS antisense oligo. Indeed, in cells treated with L-NIO only, this correction rendered NO measurement almost undetectable. Results of these studies (Figure 5) were consistent with a role for eNOS-mediated NO release in the preservation of mitochondrial mass by adenosine in the face of TNFα. Adenosine alone had no significant effect on NO levels (p = 0.17). However, 48 h incubation with TNFα (1 ng/ml) elicited an almost 40% decrease in measured NO, an effect that was reversed by adenosine. In turn, adenosine-mediated reversal of the effect of TNFα was prevented by both the NOS inhibitor, L-NIO, and transfection of cells with morpholino antisense oligo to eNOS (NOS3), while the control, reverse-sequence morpholino oligo (SON3) had no effect.

### Adenosine-elicited, NO-dependent Reversal of TNFα-induced Decrease in Mitochondrial Mass is Mediated by Soluble Guanylate Cyclase and cGMP

The stimulatory effect of NO on mitochondrial biogenesis has previously been shown in several non-endothelial cell types to be mediated by production of cGMP via soluble guanylate cyclase (sGC) [22],[30]. Since we found that adenosine’s effects in our model system appear to be mediated by NO, we next tested whether NO’s actions on TNFα-induced mitochondrial mass deficit were mediated by a sGC/cGMP-dependent mechanism. First, the potent and selective inhibitor of NO-sensitive sGC, ODQ, reversed adenosine’s effect to limit TNFα-induced decrease in mitochondrial mass. When given alone, ODQ reproduced the effect of TNFα (Figure 8). Treatment with a sGC activator, YC-1, mimicked the effect of adenosine, as did 8-Br-cGMP, a cell-permeant cGMP analog (Figure 6A). Second, ODQ reversed the attenuating effect of detaNO on TNFα-induced mitochondrial mass deficit, and both YC-1 and 8-Br-cGMP reversed TNFα’s effect in cells where eNOS expression was knocked down by the morpholino eNOS antisense oligomer (Figure 6B). Finally, when given alone, neither YC-1 nor 8-Br-cGMP produced an increase in MTG fluorescence, similar to what was observed in response to adenosine (Figure 8). These results support the hypothesis that adenosine’s effect is mediated through an NO-dependent sGC/cGMP-mediated mechanism.

### Adenosine-elicited, NO-dependent Preservation of Mitochondrial Content, Membrane Potential and Prevention of Apoptosis under Cytokine Challenge is Mediated by PGC-1α

**Figure 7:**
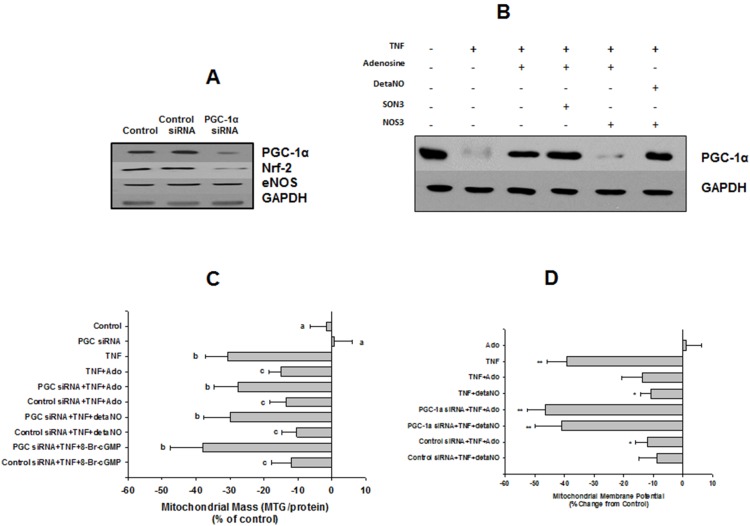
Effects of adenosine/NO are mediated by a PGC-1α-dependent mechanisim. (**A**) Western blot of expression of PGC-1α, Nrf-2, eNOS, and GAPDH, 48 h after transfection with either control or PGC-1α-specific siRNA. (**B**) Western blot of PGC-1α expression in response to TNFα±Ado or detaNO in either non-transfected cells, or cells transfected with either control (SON3) or eNOS antisense (NOS3) morpholino oligonucleotides. Blot shown is representative of 3 separate experiments. (**C**) MTG fluorescence after 48 h incubation with TNFα±Ado, detaNO, or 8-Br-cGMP in either control or PGC-1α siRNA-transfected cells (PGC siRNA). Data are from 4 separate experiments for reach group, differing letters denote significant between-group differences, p<0.05. (**D**) Measurement of Ψ in HMEC-1 cells in 24-well plates, treated as indicated, then loaded with TMRM or MTG dyes, as described in Methods. Data are means ± SEM for 4 replicates for each treatment/time combination, repeated 3 separate times. Asterisks denote values significantly different from control value, *: P<0.05, **: p<0.01.

**Figure 8:**
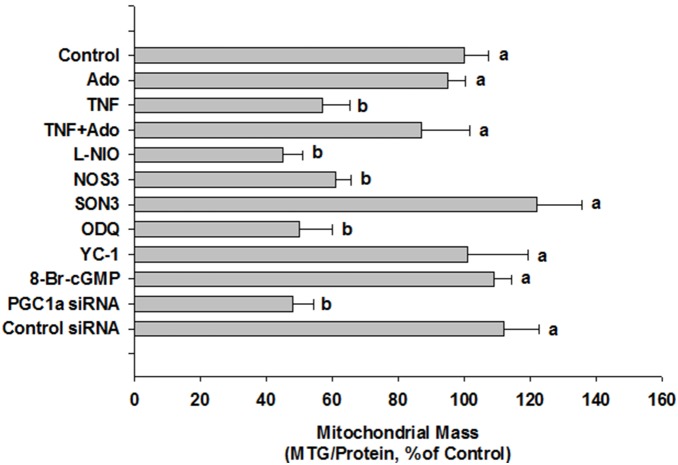
Control studies of effect of individual treatments (48 h) on MTG fluorescence. Indicated treatments were as described for other figures. Values are means ± SEM of 4 separate experiments per treatment except for NOS3/SON3 where n = 5 and PGC-1α/Control siRNA where n = 3. Differing letters denote significant between-group differences.

**Figure 9:**
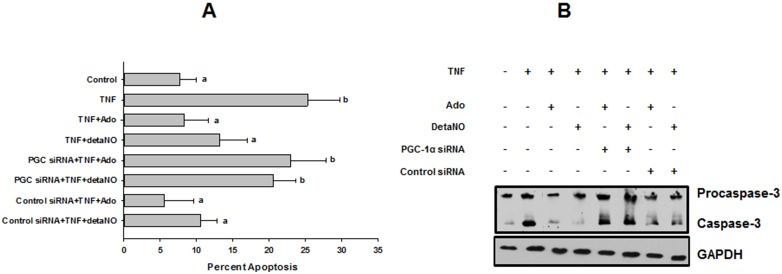
Protection of HMEC-1 cells from TNF-induced apoptosis by adenosine or detaNO is dependent on PGC-1α. (**A**) Apoptosis assayed by counting of DAPI-stained cells on glass coverslips after 72 h incubation with TNFα±Ado or detaNO in either control or PGC-1α siRNA-transfected cells (PGC siRNA). Differing letters denote significant between-group differences, experiment was repeated 3 times. (**B**) Western blot of expression of cleaved (activated) caspase-3 in cells treated similarly as in panel (A).

Our finding that TNFα decreased expression of both eNOS and PGC-1α in parallel with its effects on mitochondrial mass raised the possibility that preservation of a PGC-1α-dependent biogenesis pathway may be an obligatory downstream target of adenosine-elicited, NO-mediated protection. In addition, adenosine reversed TNFα-induced decrease in expression of PGC-1α (Figure 7B), a finding consistent with previous studies showing that NO can modulate expression and activity of PGC-1α [32]. However, TNFα may also modulate expression and/or activity of PGC-1α by NO-independent mechanisms, e.g. through stimulation of NFκB [44],[45], which would support an alternate hypothesis that adenosine’s effects on eNOS and PGC-1α are separate and independent. In order to distinguish between these two possibilities, we examined the ability of adenosine and the NO donor, detaNO to reverse TNFα’s effect on MTG fluorescence under conditions where expression of PGC-1α had been knocked down using an siRNA. Treatment of HMEC-1 cells with siRNA to PGC-1α effected an 80–90% knockdown of PGC-1α expression by 48 h post-transfection. This was associated with a 70–75% decrease in expression of Nrf-2, but no significant change in eNOS expression (Figure 7A). The ability of adenosine, detaNO, or 8-Br-cGMP to prevent TNFα-induced decreases in mitochondrial mass was reversed in cells treated with siRNA to PGC-1α, whereas the control siRNA had no effect (Figure 7C). Similarly, both adenosine and detaNO attenuated TNFα-induced decrease in mitochondrial membrane potential (Figure 7D) and increase in apoptosis (Figure 9), while neither had an effect when given alone (Figure 8). These protective effects were significantly reversed in cells in which PGC-1α expression was knocked down (Figures 7D, 9). Finally, in cells treated with the morpholino antisense construct to eNOS, adenosine was unable to reverse TNFα-induced decreases in expression of PGC-1α, but the NO donor, detaNO did rescue PGC-1α expression (Figure 7B). This supports the hypothesis that endothelial mito/cytoprotection by adenosine is mediated through preservation of NO-dependent PGC-1α expression. In summary, the data in figures 7 and 9 demonstrate 1) a consistent correlation between defense of mitochondrial mass and function, and 2) a negative association between defense of mitochondrial mass and cell survival in HMEC-1 cells.

## Discussion

Prolonged exposure (48 h) of microvascular endothelial cells to TNFα produced deficits in several markers of mitochondrial function and mass, and parallel deficits in expression of eNOS and cellular NO levels, and expression of PGC-1α, associated with a decrease in mitochondrial function and subsequent increase in apoptosis. These effects were reversed by adenosine. Our subsequent studies suggest that adenosine acts to prevent TNFα-induced decreases in mitochondrial mass and function, at least in part by blocking an inhibitory effect of the cytokine on an eNOS-PGC-1α regulatory axis for mitochondrial biogenesis. These results are the first to link adenosine with this pathway.

Numerous studies have reported TNFα-elicited, mitochondrial respiratory dysfunction [46]–[48] and cell apoptosis [49],[50], typically associated with increased production of both mitochondrial and extra-mitochondrially-derived reactive oxygen species (ROS) [48],[51]. Consistent with these previous findings, we observed time and TNFα dose-dependent decreases in mitochondrial membrane potential and cellular ATP levels, and increased apoptosis. Although the decreases in cellular ATP levels were statistically significant, they nevertheless constituted a deficit in ATP of no more than about 12–17%, roughly half that produced by inhibition of electron transport at complexes I or IV in endothelial cells [19]. At the lower dose of TNFα (1 ng/ml), we also observed a significant decrease in mitochondrial mass after 48 h incubation. At this dose of TNFα, the deficit in mitochondrial content preceded the increase in apoptosis which was not seen until 72 h.

Compared with clearly documented effects of TNFα on mitochondrial respiratory function and apoptosis, relatively few studies have specifically examined cellular mitochondrial content in response to TNFα [31],[46],[52],[53], and the results have been conflicting: some studies in adipocytes and skeletal or cardiomyocytes report TNFα-induced decreases in mitochondrial content [31],[53], one study in adipocytes reported no effect of TNFα on mitochondrial content [46], and one in HUVEC-derived EA.hy926 cells found that TNFα produced increases in both respiratory activity and mitochondrial mass [52]. Our findings are consistent with most of those reported in non-endothelial cell types [31],[53], but not with the results in EA.hy926 cells [52]. Reasons for this discrepancy are not clear, but may be due to the different cell lines used, or the differences in TNFα dose or time of exposure–our findings were obtained in cells exposed to 1 ng/ml of TNFα for 48 h, versus a 10-fold higher dose over a significantly shorter period (6 h) [52]. Our finding of a time-dependence for the effects of TNFα is consistent with the latter possibility. Overall, the results indicate a significant mitochondrial functional deficit associated with a decrease in cellular mitochondrial content in response to TNFα. Although we observed clear indication of decreased mitochondrial biogenesis, the possible contribution of TNFα-stimulated mitophagy cannot be ruled out in our studies. This has been a little-studied issue with regard to TNFα, but remains a possibility, particularly in view of recent novel findings in TNFα-stimulated macrophages [54].

Previous work in animal models has provided strong evidence that preconditioning treatments rapidly induce the release of significant amounts of adenosine [41], which then acts as a trigger for subsequent events that eventually lead to a preconditioned (i.e. protected) phenotype [2]–[4],[9],[27],[28]. A critical factor lying immediately downstream of adenosine appears to be release of nitric oxide (NO), mediated by the endothelial isoform of nitric oxide synthase (eNOS) [4]. In turn, increased NO has a number of effects, both at the level of the endothelium, as well as in vascular smooth muscle which could contribute to protection. NO-mediated promotion of mitochondrial biogenesis has been demonstrated in various cell types, including adipocytes and myocytes [22],[29]–[31]. In addition, the role of NO in mediating resveratrol-induced mitochondrial biogenesis has been demonstrated in endothelial cells [55]. Finally, TNFα has been shown to decrease eNOS-dependent mitochondrial biogenesis (31). Our measurements of cellular NO levels (Figure 5) are consistent with a role for eNOS-derived NO production in mediating the protective effects of adenosine. Overall, the results are consistent with a link between adenosine and the NO-dependent biogenesis pathway.

Interestingly, the marked effect of L-NIO (at least 80% decrease in NO) compared with eNOS knockdown suggests other possible sources of NO in HMEC-1 cells. Although the precise nature of such sources is currently undefined in our system, one possibility is the inducible isoform of nitric oxide synthase (iNOS). Expression of both eNOS and iNOS has been reported in microvascular endothelial cells from the intestine [56], and HMEC-1 cells were recently found to also express iNOS (J.S. Alexander, personal communication). However, the precise role of possibly multiple sources of NO in our model system will require further investigation. With regard to the present studies, because eNOS knockdown + TNFα in the presence of adenosine was sufficient to reproduce the effect of TNFα alone, this strongly suggests that eNOS-derived NO is sufficient to mediate the results reported herein.

Adenosine reversed both TNFα-induced deficits in PGC-1α expression and mitochondrial mass and membrane potential, as well as the increase in apoptosis. This was prevented by siRNA knockdown of PGC-1α, suggesting that adenosine’s mitoprotective effects may have been mediated by modulating TNFα-induced dysfunction in PGC-1α-dependent mitochondrial biogenesis. Our other major finding is that this PGC-1α-dependent mechanism appears to be downstream from a NO-sGC/cGMP pathway. This hypothesis is supported by 1) reversal of TNFα-induced decrease in expression of both eNOS and PGC-1α and decreased mitochondrial mass by adenosine, 2) blockade of adenosine-elicited rescue of PGC-1α expression and mitochondrial content by eNOS knockdown, 3) their rescue in the face of eNOS knockdown with either detaNO or 8-Br-cGMP, but not adenosine, and 4) the inability of adenosine, detaNO, or 8-Br-cGMP to reverse TNFα’s effect under conditions of PGC-1α knockdown.

Our proposed eNOS-PGC-1α axis for control of mitochondrial biogenesis is consistent with previous findings [30],[31],[43], and the present results indicate for the first time, that adenosine may activate this pathway in endothelial cells under conditions of inflammatory stress. Adenosine has recently been found to trigger mitophagy in cardiomyocytes [9], and this effect, presumably to promote culling of dysfunctional mitochondria, has been proposed as a mechanism underlying adenosine-elicited preconditioning in the heart. Our findings are consistent with a novel, adenosine-triggered, mitoprotective mechanism based on preservation of mitochondrial mass in endothelial cells. Further work will be required to determine whether this mechanism might contribute to adenosine-mediated preconditioning [2]–[4],[27].

The mechanism mediating preservation of eNOS-dependent NO release by adenosine in the present studies is not clear. Although adenosine increases rapid and transient phosphorylation of eNOS at Ser1177 in HMEC-1 cells, an effect dependent on 1) adenosine A2a, but not A1 receptors, and 2) ERK1/2 activation (unpublished observations), the potential role of this acute stimulation of apparent eNOS activity in the current context of mitochondrial function and biogenesis over a longer period (48 h) is unclear, and remains under investigation. Similar to our measurements of mitochondrial mass, we did not observe a stimulatory effect of adenosine alone on eNOS expression (Figure 4B). Thus, it is possible that adenosine’s specific action in our studies was to block or reverse a negative effect of TNFα on eNOS expression [31],[57]–[60]. Whether such an “anti-TNFα” effect involves inhibition of ROS release [31], is also the subject of ongoing investigation in our laboratory.

The intervening mechanism between NO-induced sGC/cGMP activity and PGC-1α in our studies is also unclear. One possibility is that NO could trigger activation of AMP kinase (AMPK) [26],[61],[62], itself known to be an activator of PGC-1α and mitochondrial biogenesis [1],[8],[61]. However, in preliminary studies, we have observed a significant increase in mitochondrial mass in HMEC-1 cells treated with the AMPK inhibitor, compound C (unpublished observations), indicating an unexpected complexity in the potential role of AMPK in mitochondrial biogenesis in endothelial cells. A similarly unexpected increase in mitochondrial mass in response to treatment with compound C was recently reported in T cells undergoing T cell receptor activation [63]. Thus, further work will be required to clarify a possible role of AMPK in our endothelial model of mitochondrial biogenesis.

Regulation of mitochondrial biogenesis is but a single aspect of the functions of the transcriptional co-activator, PGC-1α. This key molecule plays a broad, pleiotropic regulatory role in overall cellular energy metabolism and cell defense that extends well beyond simply regulation of mitochondrial content, including regulation of oxidative fuel consumption [35] and expression and activity of ROS defense mechanisms [32],[64]. If PGC-1α-mediated protection plays a role in preconditioning strategies, it seems likely that such a role is multifactorial, and not limited to mitochondrial biogenesis. Thus, our finding that PGC-1α is necessary for adenosine’s ability to preserve endothelial mitochondrial mass and prevent apoptosis in the face of TNFα challenge does not rule out other potential PCG-1α-dependent cytoprotective mechanisms. Additional insight into the potential role of mitochondrial biogenesis *per se* in preconditioning might be gained by examining factors further downstream from PGC-1α in the specific mitochondrial biogenesis control pathway.
