# Correction: Adenosine Prevents TNFα-Induced Decrease in Endothelial Mitochondrial Mass via Activation of eNOS-PGC-1α Regulatory Axis

**DOI:** 10.1371/journal.pone.0103692

**Published:** 2014-07-21

**Authors:** The PLOS ONE Staff

## Notice of Republication

This article was republished on July 1st, 2014, due to panel B of Figure 3 being erroneously removed during the production process. The publisher apologizes for this error. Please download the PDF again to view the corrected article and the complete Figure 3. The originally published, uncorrected article and the republished, corrected article are provided here for reference.

## Supporting Information

File S1 Originally published, uncorrected article
(PDF)

File S2 Republished, corrected article
(PDF)
